# Disentangling Drivers of Soil Bacterial and Fungal Diversity on Tropical Islands

**DOI:** 10.1002/ece3.72548

**Published:** 2025-11-23

**Authors:** Yikang Cheng, Yawen Huang, Run Zhang, Shurong Zhou

**Affiliations:** ^1^ School of Ecology Hainan University Haikou China; ^2^ School of Tropical Agriculture and Forestry Hainan University Haikou China

**Keywords:** bacterial, fungal, growth strategies, island biogeography, plant‐microbe interactions, soil pH

## Abstract

Island area and variations in climate (e.g., temperature and precipitation) are widely known to affect the insular‐dwelling soil bacterial and fungal communities. Such effects can be context‐dependent, and many factors can determine the diversity of soil microbes. For example, island area and climate factors can directly influence soil bacterial and fungal communities, as well as exert an indirect effect by altering plant communities and/or soil properties. However, we lack a comprehensive mechanistic understanding of the relative importance of these potential drivers. To explore the key factors affecting insular soil microbial community dynamics, we selected 20 representative tropical islands in the South China Sea and established two to eight permanently marked plots based on the island area. Then, we investigated the plant community composition and measured growth strategy‐related plant traits (i.e., specific leaf area and leaf dry matter content). Concurrently, we analyzed a series of soil properties (i.e., pH, salinity, organic carbon, total nitrogen, total phosphorus, total potassium, and carbon/nitrogen ratio) and the diversity of soil bacterial and fungal communities. For the soil bacterial community, based on structural equation modeling (SEM) analysis, we found an indirect effect of climate factors and island area on bacterial richness through their influence on plant richness, in addition to the direct effect of island area on bacterial richness. In contrast, for the soil fungal community, we found that the influences of temperature and precipitation are primarily indirect via changes in soil pH and the community‐weighted mean (CWM) value of plant leaf dry matter content. Overall, this study highlights that island‐dwelling soil bacterial and fungal communities are shaped by island‐specific plant community and soil pH, which may interplay with macroscopic threats like ongoing sea level rise and biological invasions, thus providing crucial insights into the dynamics of soil microorganisms under global change scenarios.

## Graphical abstract

Island area and variations in climate (e.g., temperature and precipitation) are widely known to affect the insular‐dwelling soil bacterial and fungal communities. However, we lack a mechanistic understanding of the relative importance of the potential drivers. Overall, this study highlights the importance of plant richness, leaf dry matter content, and soil pH in shaping the patterns of island‐dwelling soil bacterial and fungal communities.

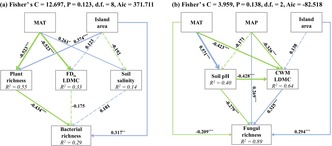

## 1 Introduction

Soil bacteria and fungi, the main components of below‐ground microorganisms, play a vital role in many ecological processes and maintain the functioning of terrestrial ecosystems (Bardgett and van der Putten 2014). Currently, ongoing global change, including habitat loss and variations in climatic factors (e.g., temperature and precipitation), has significantly altered the diversity patterns of soil bacterial and fungal communities (Wu et al. 2022; Baldrian et al. 2023). Although the impact of global change on soil microbes has been widely investigated across various ecosystems (Yu et al. 2024), the underlying mechanisms have not been systematically explored, as many factors may contribute to such changes. In general, changes in available habitat area and climate conditions could directly influence soil bacterial and fungal communities as well as through indirect effects, including altering abiotic soil environments (Glassman et al. 2017; Wang and Kuzyakov 2024) and/or biotic factors such as plant diversity (Dassen et al. 2017; Wang et al. 2022) and plant growth strategies related traits (Bergmann et al. 2020; Prada‐Salcedo et al. 2021). Therefore, distinguishing the relative importance of these drivers will be crucial for understanding the dynamics of the soil microbial community in the context of ongoing habitat loss and climate change.

Island ecosystems, characterized by their varying sizes and unique climatic conditions, provide a useful backdrop for distinguishing the direct and indirect effects of habitat size (i.e., island area) and climate variation on soil bacterial and fungal communities (Helmus et al. 2014; Whittaker et al. 2017; Li et al. 2020). On the one hand, larger islands tend to have a higher colonization rate and a lower extinction rate (Connor and McCoy 1979), island area would directly increase soil bacterial and fungal diversities (Wang et al. 2020; Zheng et al. 2021; Zhou et al. 2024); On the other hand, island area could also indirectly influence soil bacterial and fungal diversities by altering soil properties (Li et al. 2020; Zhou et al. 2024) and/or plant community (Zheng et al. 2021; Xu, Yang, et al. 2023). Plants are widely perceived to influence soil bacterial and fungal communities in various ways (Bardgett and Wardle 2010), including through root exudates and community productivity (Badri and Vivanco 2009; Schlatter et al. 2015). Additionally, plant identity can shape their associated microbial communities, and a long‐term biodiversity experiment found that soil bacterial richness was higher in legume plants than in small herb plants (Dassen et al. 2017). Analogously, changes in climatic conditions would also directly select those soil bacterial and fungal taxa that can adapt to such environments, as well as exert an indirect effect through altering soil properties and plant community (Yu et al. 2024). Indeed, accumulated evidence suggests a strong link between aboveground plant diversity and soil bacterial or fungal diversity (Dassen et al. 2017; Porazinska et al. 2018). Several large‐scale studies have also demonstrated that the richness of plant community and soil‐inhabiting microbes is strongly correlated across various ecosystems (Tedersoo et al. 2014; Liu et al. 2020; Wang et al. 2022). Furthermore, in addition to the indirect effect through affecting plant richness, plant functional traits, which are related to growth strategies, also have a potential influence on soil bacterial and fungal communities (Wardle et al. 2004; Bardgett 2017; Prada‐Salcedo et al. 2021).

Island area and variations in climate conditions (i.e., temperature and precipitation) can indirectly influence soil bacterial and fungal communities through altering soil properties (i.e., soil‐mediated effects) (Wang et al. 2020; Zhou et al. 2020, 2024; Yu et al. 2024). Specifically, larger islands tend to provide a more favorable abiotic environment (e.g., nutrient‐rich soil and suitable soil pH) than smaller islands (Ewers and Didham 2006). Therefore, larger islands may be able to harbor more soil microbe taxa than smaller islands. For example, the greater taxonomic diversity of soil fungal and bacterial communities in larger tropical oceanic islands was significantly driven by the variation in soil pH (Zhou et al. 2024). Likewise, soil total phosphorus content and moisture have been shown to be key factors influencing the response of soil bacterial and fungal communities to altered precipitation regimes in meadow steppe ecosystems (Yang et al. 2021). Additionally, a meta‐analysis also indicated that global warming would significantly increase both soil bacterial diversity and fungal richness through altering soil organic carbon content (Xu, Huang, et al. 2023).

Island area can also alter plant richness and functional trait composition (Ottaviani et al. 2020; Schrader et al. 2021; Ferreira‐Arruda et al. 2022), as can changes in temperature and precipitation (Franklin et al. 2016; Aguirre‐Gutiérrez et al. 2025). These changes can then indirectly alter soil bacterial and fungal communities via plant–microbe interactions (i.e., plant‐mediated effects) (Yu et al. 2024). Indeed, high‐diversity plant communities with more diverse litter and root exudates can support a greater number of microbial taxa by enhancing resource availability and altering physical microhabitats (Millard and Singh 2010; Steinauer et al. 2015). Besides, resource‐conserving plant species with low relative growth rates (e.g., low specific leaf area [SLA] and high leaf dry matter content [LDMC], which were core components of the leaf economics spectrum (Wright et al. 2004)) produce low‐quality litter that promotes the growth of fungal relative to bacterial communities compared to faster‐growing exploitative plant species that tend to favor soil bacterial communities (Wardle et al. 2004; Bardgett 2017). Another empirical case study has also found that plant communities dominated by resource‐exploiting species in grassland ecosystems have bacterial‐dominated microbial communities, while dominance by conservative plant species is associated with fungal‐dominated microbial communities (de Vries et al. 2012). Likewise, the soil fungal community in forest ecosystems is also significantly correlated with plant litter and absorptive root traits (Prada‐Salcedo et al. 2021). Therefore, examining the relative importance of the direct effect of island area and climate factors, the soil‐mediated effect, and the plant‐mediated effect will allow us to explore the specific drivers of soil microbial community dynamics.

Tropical islands, with their unique environmental context and rich biodiversity, provided an ideal place to test the relative importance of the aforementioned drivers on insular‐dwelling soil bacterial and fungal diversities (Zhou et al. 2024). Here, we conducted a large‐scale investigation to distinguish the effects of multiple potential drivers using 20 tropical islands widely distributed in the South China Sea (the distance between islands varies from several kilometers to several hundred kilometers) as a model system. We tested the following hypotheses: (i) Island area and climate factors (i.e., temperature and precipitation) influence soil microbial diversity, but their effects differ for soil bacterial and fungal communities; (ii) These effects are both direct and indirect, mediated by changes in (a) plant species richness, (b) plant functional composition (e.g., community‐weighted means of specific leaf area and leaf dry matter content), and (c) soil properties (e.g., pH, organic matter content) (Figure S1). By simultaneously considering changes to below‐ground soil conditions and above‐ground plant communities, we aimed to provide a deeper mechanistic understanding of the biogeography of island‐dwelling soil microbes.

## 2 Materials and Methods

### 2.1 Study Sites and Sampling

We selected 20 islands with minimum levels of human disturbance in the South China Sea that varied in area (1.19–405.99 ha), mean annual temperature (24.57°C–27.05°C), mean annual precipitation (1340–1616 mm), and vegetation composition (Table S1; Figure S2). On each island, we established two to eight permanently marked 20 m × 20 m plots, with the number of plots surveyed per island approximately proportional to the log‐transformed area of the island, resulting in a total of 82 plots across all islands. To minimize the influence of spatial autocorrelation among the plots, we ensured that the spatial distance between any two adjacent plots was greater than 100 m (except for the smallest island, where the sample plots were at least 40 m apart).

Due to the absolute dominance of woody plants (e.g., trees and shrubs) on tropical islands, we identified and enumerated all woody plants rooted within the plots with a diameter at breast height (DBH) of ≥ 1 cm (Martinez‐Ramos and Alvarez‐Buylla 1998; Zhou et al. 2024); a total of 261 plant species were identified across all islands. For soil bacterial and fungal sampling, we established three evenly distributed 2 m × 2 m quadrats along the diagonal of each larger sample plot and collected four randomly distributed soil cores (15 cm in depth) within each quadrat. We then combined and homogenized all samples within each plot, resulting in a total of 82 composite soil samples across the islands. We sieved the composited soil samples through a 2 mm mesh to remove large particles and divided each into two subsamples. We kept one subsample at 4°C to determine the soil properties and the other at −20°C for later DNA extraction.

### 2.2 Plant Trait Measurements

For each plant species within each plot, we randomly selected three to five adult individuals for trait measurements, resulting in a total of 3045 plant individuals being collected for trait measurements. Briefly, three healthy and mature leaves of each individual were selected to measure the fresh leaf weight (g) and leaf area (LA, mm^2^) using the scanner and LA‐S Leaf Area Analysis software (Wseen Detection Technology Co. Ltd., Hangzhou, China). Then, specific leaf area (SLA) and leaf dry matter content (LDMC) were calculated after drying the leaves at 65°C to a constant mass and weighing them to 0.0001 g.

### 2.3 Soil Parameters

The study area was characterized by soils typical of tropical islands. According to the World Reference Base (WRB) classification, the predominant soil types were Xanthic Ferralsols, Arenosols, and Ferralic Cambisols. We measured seven soil properties that could influence soil microbial communities. Briefly, we measured soil pH with a pH analyzer (Metter‐S210 SevenCompact, Switzerland); we measured soil organic carbon (SOC) and total nitrogen (STN) content using an element analyzer (Elementar Vario EL III, Hanau, Germany); soil total phosphorus (STP) using the Olsen colorimetric method on a UV–Visible Spectrophotometer; soil total potassium (STK) by extracting with 1 M ammonium acetate and analyzing using Inductively Coupled Plasma‐Optical Emission Spectrometry (ICP‐OES); and soil salinity (g/kg) was measured as total dissolved solids content by evaporating a filtered volume of water to dryness and weighing the residual solids. We also calculated the soil C:N ratio using SOC and STN.

### 2.4 Climate Data

We sourced climate data based on the geographical coordinates (i.e., longitude and latitude) of each island from the WorldClim Global Climate and Weather Data (http://www.worldclim.org). Specifically, we downloaded the mean annual temperature (hereafter referred to as temperature) and mean annual precipitation (hereafter referred to as precipitation) data for each island, as many previous studies have shown that temperature and precipitation are important factors affecting soil bacterial and fungal communities (Wu et al. 2022; Yu et al. 2024). Additionally, due to the strong correlation between island precipitation and isolation (*p* < 0.001; *R* ^2^ = 0.849; Figure S3), we excluded the effect of isolation from subsequent analyses.

### 2.5 Molecular and Bioinformatics Analysis

We extracted DNA from all soil samples using the Magnetic Soil DNA Extraction Kit (DC306‐09, FINDROP). For bacteria, we targeted the V4 region of the 16S ribosomal RNA (rRNA) gene, using 515‐F and 806‐R primer pairs. For fungi, we targeted the second nuclear ribosomal internal transcribed spacer (ITS2) region of the rRNA operon using the ITS3 and ITS4 primer pairs. Polymerase chain reaction (PCR) amplification was done with BioRad S1000 (Bio‐Rad Laboratories, CA). Sequencing was conducted by the ‘Magigene’ company (Guangzhou, China). After we collected sequencing data, we applied strict quality control steps. Briefly, we re‐assigned the raw sequences to the samples in Mothur (V1.35.1) (Schloss et al. 2009) and merged paired‐end clean reads with the help of FLASH (V1.2.11) (Mago and Salzberg 2011). Then, low‐quality sequences, with an average quality score < 25 and length < 200 bp, were discarded, and the remaining high‐quality sequences were trimmed using Trimmomatic (V0.33) to obtain high‐quality clean sequences (Bolger et al. 2014). During clustering, USEARCH was used to remove chimeric sequences. The remaining non‐chimeric sequences were clustered into operational taxonomic units (OTUs) at a 97% similarity level. All the bacterial and fungal OTUs were searched against the SILVA and UNITE databases. We then removed OTUs that were not classified as either bacterial or fungal, resulting in a total of 48,554 bacterial OTUs and 32,116 fungal OTUs. Prior to statistical analyses, the community data were rarefied using the function ‘*rarefy_even_depths*’ in the ‘*phyloseq*’ package.

### 2.6 Plant and Soil Microbial Diversity

We measured plot‐level taxonomic richness as an indicator of plant and soil microbial diversity. We also calculated (1) the functional dispersion (FD_is_) of SLA and LDMC and (2) the community‐weighted mean value (CWM) of SLA and LDMC as indicators of plant functional trait diversity using the average trait value weighted by species abundance. We calculated both of these values using the function ‘*dbFD*’ in the ‘*FD*’ package (Laliberté and Legendre 2010).

### 2.7 Statistical Analyses

#### 2.7.1 Effect of Climate and Island Area on Plant Diversity, Soil Properties, Bacteria, and Fungal Diversities

Linear models were applied to test the effects of temperature, precipitation, and island area on soil characteristics, plant species richness, plant functional diversity (i.e., FD_is_ and CWM of SLA and LDMC), as well as the taxonomic richness of soil bacterial and fungal communities.

#### 2.7.2 Relative Importance of Abiotic and Biotic Factors on Soil Microbial Diversity

Linear models were applied to test the effects of each abiotic (i.e., seven soil variables) and biotic factor (i.e., plant richness and functional diversity) on bacterial and fungal richness. Then, we calculated the standardized coefficient and the coefficient of determination. In addition, we treated the seven soil properties, plant richness, and functional diversity as predictor variables that influence the diversities of soil bacteria and fungi. We estimated the importance of the variables, as well as the variance explained by the models, using the ‘*lmg*’ function in the R package ‘*relaimpo*’ (Grömping 2006).

#### 2.7.3 Direct and Indirect Effects of Climate and Island Area on Soil Microbial Diversity

The analysis to distinguish the direct and indirect effects was performed following the methods described by Cheng et al. (2023). First, we conducted a Random Forest analysis to determine those variables that have strong explanatory power on microbial diversity (Leo 2001). The Random Forest method is particularly effective for making predictions and can accommodate both linear and nonlinear relationships (Strobl et al. 2007). The eight most important variables (i.e., those that most effectively minimized mean squared error (MSE) during random permutation testing) were prioritized as candidate predictors in subsequent analysis (Figure S4). We estimated Random Forest analysis using the ‘*randomForest*’ function in the ‘*randomForest*’ package (Liaw and Wiener 2002).

Then, we conducted model selection to identify key predictive variables that significantly influence the soil bacterial and fungal communities, respectively. To avoid model overfitting, pairwise Pearson correlations between variables were checked, and one variable was removed from pairs with high correlations (*r* > 0.7) (Figure S5). Further, we used the ‘*dredge*’ function in the ‘*MuMIn*’ package to generate a subset of candidate models derived from the full model. These models were ranked by the Akaike information criterion (AICc) and corrected for small sample sizes (Barton 2014). Models differing by < 2 AICc units were considered equally plausible.

Finally, based on the model selection result (Table 1), we applied the structural equation modeling (SEM) to distinguish the direct and indirect effects of climate and island area on bacterial and fungal diversities according to the hypothesized mechanisms (Figure S1). To simplify the hypothesized model, based on the model selection results, we performed confirmatory path analysis based on the directional separation test (d‐separation test) (Shipley 2009, 2013). To improve the model fit, we simplified the initial SEM by stepwise removing the non‐significant pathways. The model was deemed an adequate fit when the model had a Fisher's *C* statistic with a *p* value > 0.05. We considered the model with the lowest AICc value as the final model. We calculated the SEM model using the ‘*psem*’ function in the ‘*piecewiseSEM*’ package (Lefcheck 2016).

**TABLE 1:** Model selection results for abiotic and biotic sources of variation of soil bacterial and fungal richness. Only models with ΔAICc < 2 are presented.

Group	Predictor variables	LogLik	AICc	△AICc	*R* ^2^
Bacteria richness	~ Plant richness + Soil salinity	−49.734	107.988	0	0.203
	~ Plant richness + FD_is_ LDMC	−49.819	108.157	0.169	0.201
	~ Plant richness + FD_is_ LDMC + Soil salinity	−48.684	108.158	0.170	0.223
	~ Plant richness	−51.009	108.325	0.337	0.177
	~ Plant richness + FD_is_ LDMC + STP	−49.118	109.025	1.037	0.214
	~ Plant richness + FD_is_ LDMC + Soil salinity + STP	−48.185	109.490	1.502	0.232
	~ MAT + Plant richness + Soil salinity	−49.409	109.608	1.620	0.209
	~ CWM LDMC + Soil salinity	−50.571	109.662	1.674	0.186
	~ MAT + Plant richness	−50.599	109.717	1.729	0.186
	~ Plant richness + STP	−50.605	109.729	1.741	0.185
	~ Plant richness + Soil salinity + STP	−49.476	109.742	1.753	0.208
	~ Plant richness + Soil salinity + Soil pH	−49.517	109.823	1.835	0.207
	~ MAP + Plant richness + Soil salinity	−49.597	109.983	1.994	0.205
Fungi richness	~ MAP + MAT + Island area + CWM LDMC + SCN + Soil pH	33.701	−49.429	0	0.896
	~ MAP + MAT + Island area + CWM LDMC + Soil pH	32.360	−49.207	0.222	0.892

Abbreviations: ∆AICc, difference between the AICc of a given model and that of the best model; AICc, akaike information criterion for small samples; CWM, community weighted mean; FD_is_, functional dispersion; LDMC, leaf dry matter content; MAP, mean annual precipitation; MAT, mean annual temperature; SCN, soil carbon/nitrogen ratio; SLA, specific leaf area; SOC, soil organic carbon content; STK, soil total potassium content; STN, soil total nitrogen content; STP, soil total phosphorus content.

## 3 Results

### 3.1 Response of Soil Microbe Diversity to Abiotic and Biotic Variables

**FIGURE 1:**
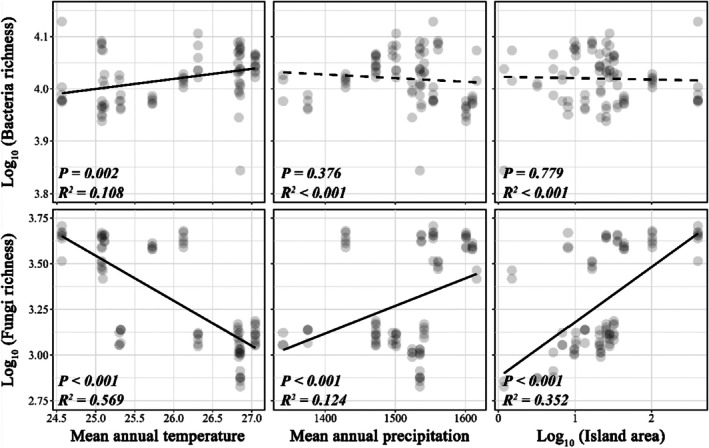
Effect of climate variables and island area on soil bacterial richness and fungal richness. Each point represents one individual sample plot. Significant correlations (*p* < 0.05) are marked as solid lines and insignificant correlations (*p* > 0.05) are marked as dashed lines.

**FIGURE 2:**
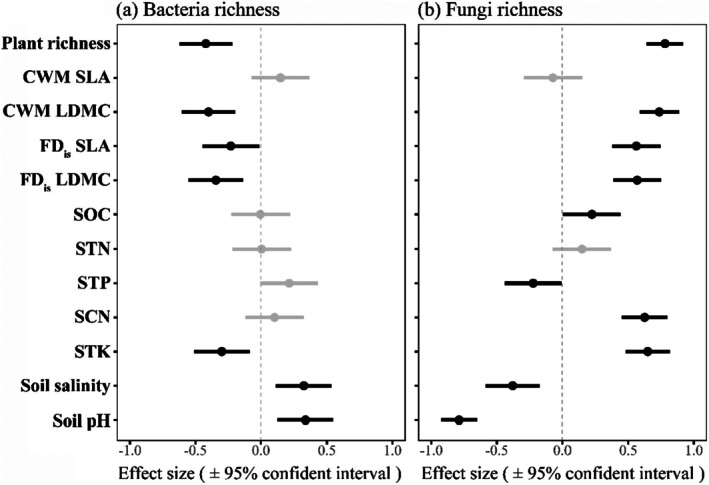
Effects of abiotic and biotic variables on soil bacterial (a) and fungal (b) richness. Effect sizes are standardized coefficients from linear models estimated separately for each predictor variable. The lines indicate a 95% confidence interval, black circles indicate significant effects and gray circles indicate non‐significant effects. CWM, community weighted mean; FD_is_, functional dispersion; LDMC, leaf dry matter content; SCN, soil carbon/nitrogen ratio; SLA, specific leaf area; SOC, soil organic carbon content; STK, soil total potassium content; STN, soil total nitrogen content; STP, soil total phosphorus content.

When we examined the influence of island area, temperature, and precipitation on soil microbial diversity, we found distinct responses of the soil bacterial and fungal communities. Specifically, bacterial richness increased with temperature but was not associated with precipitation and island area (Figure 1a), whereas fungal richness significantly decreased with temperature and increased with both precipitation and island area (Figure 1b). Likewise, for the impact of soil properties and plant community, we found that the soil bacterial richness was significantly increased with soil pH and salinity but decreased with soil total potassium content (STK) and several plant diversity variables (e.g., plant richness, community‐level weighted mean value [CWM] and functional dispersion [FD_is_] of leaf dry matter content [LDMC]) (Figure 2a). Alternatively, for soil fungal community, we found that fungal richness was significantly increased with plant diversity (e.g., plant richness, CWM and FD_is_ of LDMC), and several aspects of soil factors (e.g., soil organic carbon content [SOC] and STK), while soil total phosphorus content (STP), salinity and pH were negatively associated with fungal richness (Figure 2b).

**FIGURE 3:**
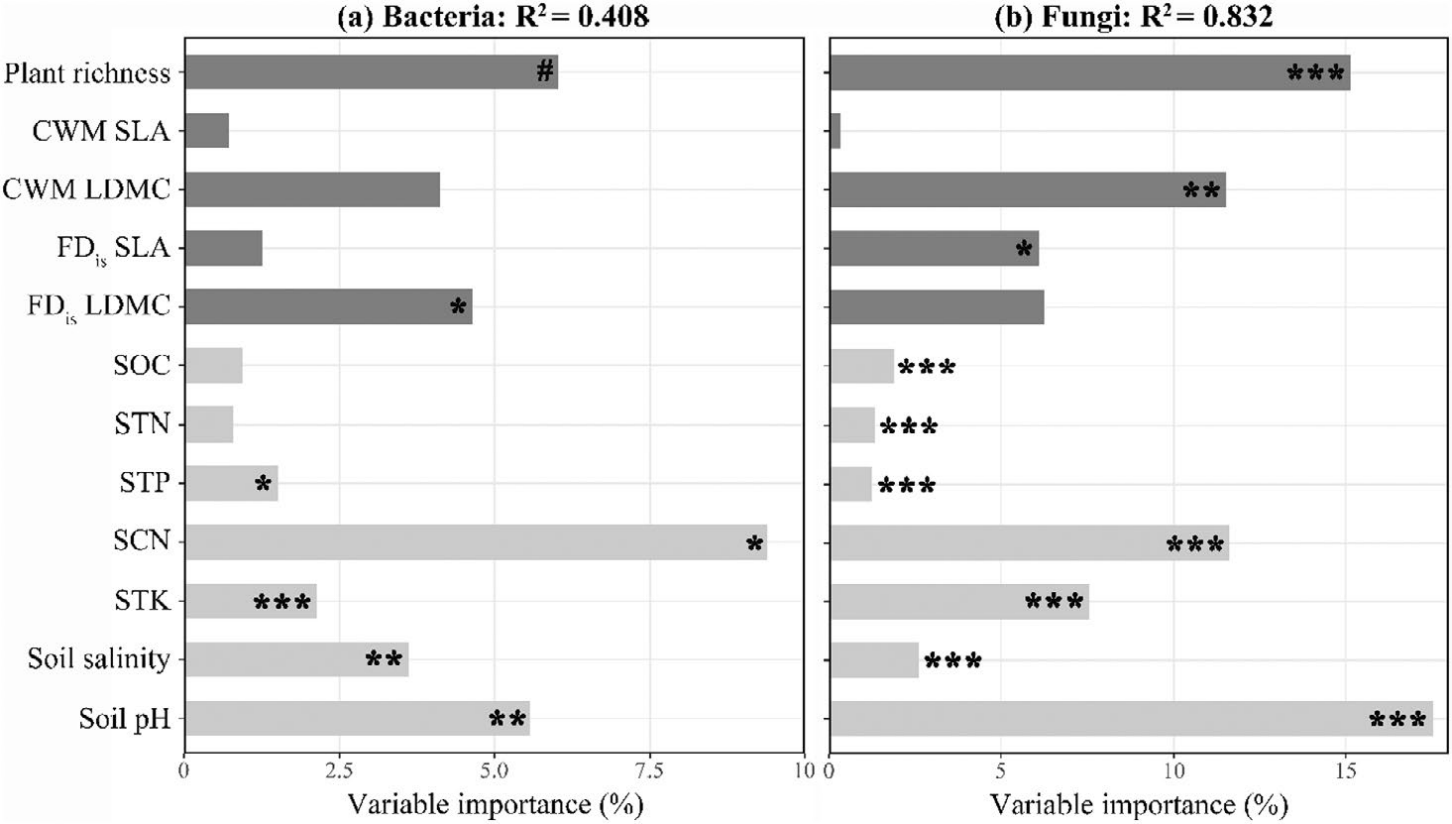
The fraction of variation in soil bacterial (a) and fungal (b) richness explained by abiotic and biotic variables. CWM, community weighted mean; FD_is_, functional dispersion; LDMC, leaf dry matter content; SCN, soil carbon/nitrogen ratio; SLA, specific leaf area; SOC, soil organic carbon content; STK, soil total potassium content; STN, soil total nitrogen content; STP, soil total phosphorus content. Significance: ***p < 0.001; **p < 0.01; *p < 0.05; ^#^ *p* < 0.10.

The variance partitioning result revealed that plant community and soil properties accounted for ~41% of the variation in bacterial taxonomic richness. The soil carbon/nitrogen ratio (SCN) and pH, as well as plant richness, were the most important predictors, explaining more than 5% of the variation (Figure 3a). For the soil fungal community, plant diversity and soil properties explained ~83% of the variation (Figure 3b), with plant richness, CWM of LDMC, soil pH and the SCN being the most important predictors, explaining more than 10% of the variation (Figure 3b).

### 3.2 Response of the Plant Community and Soil Properties to Island Area and Climate Factors

Based on the linear models, we found that plant richness, CWM of LDMC, FD_is_ of specific leaf area (SLA) and LDMC, as well as several soil properties (i.e., SOC, SCN and STK), were significantly decreased with temperature. In contrast, CWM of SLA, STP, salinity and pH were significantly increased with temperature. Likewise, we found that soil total nitrogen content and pH were positively related to precipitation, but there were no changes in plant taxonomic and functional diversities. Besides, variations in the island area, led to significant positive changes in plant richness, CWM of LDMC, FD_is_ of SLA and LDMC, as well as SCN, while soil salinity and pH significantly decreased with island area (Table S2).

### 3.3 Direct, Plant‐Mediated and Soil‐Mediated Indirect Effects of Climate Factors and Island Area on Soil Bacterial and Fungal Diversities

**FIGURE 4:**
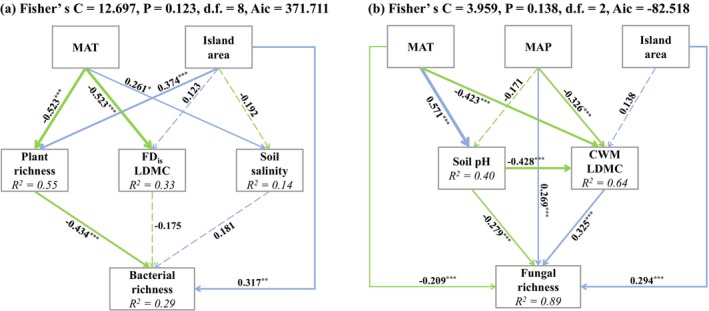
Structural equation model (SEM) results showing the pathways of climate and island area that influence soil bacterial (a) and fungal (b) richness through the aboveground plant community and/or belowground soil properties. The coefficients are standardized prediction coefficients for each causal path. Solid lines represent significant relationships (*p* < 0.05), while dashed lines represent non‐significant relationships. Blue lines represent positive effects, and green lines represent negative effects. Line thickness is proportional to the strength of the relationship. Numbers above and below arrows are standardized path coefficients (significance: ***p < 0.001; **p < 0.01; *p < 0.05). *R* ^*2*^ represents the proportion of variance explained for each dependent variable. CWM, community weighted mean; FD_is_, functional dispersion; LDMC, leaf dry matter content; MAP, mean annual precipitation; MAT, mean annual temperature.

Improved from the initial SEM (Figure S6), for bacterial richness, the best fitting SEM showed that, in addition to the direct effect of island area, temperature and island area would indirectly influence bacterial richness through altering plant richness (Fisher's *C* = 12.697, *p* = 0.123, df = 8, AIC = 371.711, Figure 4a). For the fungal community, temperature, precipitation and island area exerted a direct effect on fungal richness (Fisher's *C* = 3.959, *p* = 0.138, df = 2, AIC = −82.518, Figure 4b), and we also found an indirect effect of temperature and precipitation on fungal richness mediated by their influence on soil pH and the CWM of LDMC (Figure 4b).

## 4 Discussion

In this study, we attempt to distinguish between direct, soil‐mediated, and plant‐mediated effects of island area and climate variation on the insular‐dwelling soil bacterial and fungal communities, thereby extending previous studies to explore the specific drivers of soil microbe community dynamics in tropical island ecosystems. We observed a shift in plot‐level soil bacterial diversity, primarily driven by changes in plant species richness across the islands, which points to the importance of plant‐microbe interactions in shaping the pattern of bacterial diversity. Likewise, direct effects of climate and island area, as well as indirect effects via their influence on soil pH and plant traits, were proven to be the main driving factors of soil fungal richness, which suggests that soil properties and plant growth strategies played a more important role than plant diversity in driving the dynamics of the soil fungal community.

Consistent with our first hypothesis, we found that elevated temperature significantly increased bacterial richness while decreasing fungal richness (Figure 1), which is consistent with the findings of many previous studies that increased temperature would decrease soil fungal diversity and fungal‐to‐bacterial ratios (Pietikäinen et al. 2005; Guo et al. 2018; Feng et al. 2022). Unsurprisingly, soil bacteria and fungi tend to have opposite responses to temperature due to their different sensitivities to shifts in environmental conditions (Shen et al. 2020). Likewise, higher temperatures stimulate microbial decomposition of soil organic matter by diminishing the physical protection of soil aggregates, thereby increasing the relative growth rate of the soil bacterial community (Metze et al. 2024). Similarly, we also found a positive effect of precipitation on fungal diversity, but not bacterial diversity, as has been observed in subtropical ecosystems (He et al. 2017) (Figure 1), which suggests that soil fungal communities are more sensitive to variation in precipitation than bacteria. Contrary to our result, many studies have found that soil bacteria typically respond faster than fungi to changes in soil water availability due to their distinct physiological properties and survival strategies (Engelhardt et al. 2018; Yang et al. 2021). The possible explanation is that soil fungal taxa may preferentially live in large pores, which are filled at high moisture conditions but empty at low moisture, whereas soil bacterial taxa tend to live in smaller pores, better protected against the shift in precipitation among islands (Kaisermann et al. 2015). Besides, consistent with many previous works (Li et al. 2020; Zhou et al. 2024), we found an increase in fungal richness with island area, while island area did not affect bacterial richness (Figure 1). The potential reason for this pattern could be the opposing influence of the direct positive effect of island area on bacterial richness that was countered by the negative effect of plant richness on bacterial richness, as indicated by the structural equation modeling analysis (Figure 4a).

In support of our second hypothesis, we found that the island area and temperature significantly affected soil bacterial richness mainly through indirectly influencing plant richness (Figure 4a). On the one hand, islands with higher plant diversity can regulate soil bacterial diversity by providing more heterogeneous litter and microhabitat types, higher levels of resources, ecological niche differentiation, or host specificity (Wardle et al. 2004; Bardgett and Wardle 2010; Gould et al. 2016); on the other hand, a manipulating experiment showed that there was a significant negative relationship between bacterial and plant species richness, which was mediated by the greater concentrations of soil resources and aboveground plant biomass along with the plant richness gradient (Schlatter et al. 2015). In general, higher bacterial diversity was associated with a greater proportion of antagonistic bacteria. However, highly diverse plant communities have both substantially greater soil resource concentrations and smaller proportions of antagonistic bacteria, resulting in less diverse soil bacterial communities (Bakker et al. 2013). Additionally, it is possible that specific factors simultaneously regulate the diversity of plants and microbes. For example, Wang et al. (2016) found that aboveground herb diversity was coupled with soil prokaryotic diversity due to their similar responses to soil pH for forest ecosystems of eastern China (Wang et al. 2016).

For the soil fungal community, we found that temperature and precipitation altered fungal richness primarily by altering soil pH and the community‐weighted mean (CWM, plot‐level trait values weighted by plant species abundance) of leaf dry matter content, which provided clear evidence that both soil conditions and plant growth strategy mediate the soil fungal community dynamics in response to climate change. Indeed, as proposed by the leaf economics spectrum, leaf dry matter content is an indicator of plant growth strategy (Wright et al. 2004), and slow‐growing plants with higher leaf dry matter content tend to produce low‐quality litter, which promotes the growth of fungi relative to bacteria (Bardgett 2017; Bergmann et al. 2020; Prada‐Salcedo et al. 2021). Besides, soil pH is known to influence the soil fungal community at both local (Glassman et al. 2017) and global (Tedersoo et al. 2014) scales. Soil pH has been proven to be an indicator of soil nutrient availability, and the exchange capacity of anions and cations in the soil is directly affected by pH (Hartemink and Barrow 2023). Further, soil pH has been referred to as the “master soil variable” that affects many soil biological, chemical, and physical properties (Minasny et al. 2016). Additionally, it is essential to acknowledge that, due to the intricate relationships between soil pH and other chemical and physical properties, some unmeasured soil indicators may also play a significant role in shaping soil microbial community dynamics.

Notably, we also found a direct effect of both climate and/or island area on the soil bacterial and fungal communities. The most invoked explanation is that temperature and precipitation may directly select the specific bacterial and fungal taxa that can persist under those conditions. For instance, the shift in precipitation could directly influence the soil microbial community by changing soil water availability (Ren et al. 2017), and temperature would select for the survival of temperature‐tolerant species and stimulate their activity in soil nutrient cycling (Guo et al. 2018). Likewise, larger islands tend to have higher species colonization rates, lower extinction rates, as well as greater microhabitat heterogeneity (MacArthur and Wilson 1967), leading to a significant direct effect of area on the soil microbial community.

In addition to these aforementioned drivers, the result of variation partitioning analysis indicated that all the considered variables explained ~41% of the variation in the soil bacterial community and ~83% in the soil fungal community (Figure 3), which indicates that other potential factors, such as plant root traits, may also regulate the diversity pattern of soil bacterial and fungal communities (Legay et al. 2014; López‐Angulo et al. 2020). For example, some root functional traits, such as specific root length and nitrogen content, are also important indicators of plant growth strategies (Reich 2014; Kong et al. 2019) and would exert potential effects on the soil microbial community. Furthermore, root physical and chemical traits can influence the composition and activity of the soil microbial community (Gillespie et al. 2021; Spitzer et al. 2021; Hennecke et al. 2025). Therefore, incorporating above‐ground leaf traits and below‐ground root traits in future studies may provide new insights into how island‐dwelling soil microbe communities respond to ongoing climate change and habitat loss.

## 5 Conclusion

Although an increasing number of studies have endeavored to examine the island biogeography of soil bacterial and fungal communities, few have explicitly disentangled the underlying driving mechanisms that lead to these patterns, especially in tropical island ecosystems. Here, by distinguishing the relative importance of the direct effect of island area and climate factors, plant‐mediated effect, and soil‐mediated effect in driving the patterns of island‐dwelling soil bacterial and fungal communities, we found that island temperature and area affected soil bacterial richness mainly through influencing plant richness, which provides solid field‐based evidence that plant community richness plays a predominant role in driving the diversity of soil bacterial communities on tropical island ecosystems. In contrast, soil fungal diversity is primarily influenced by soil pH and plant growth‐related traits. Overall, our study provides new evidence for the role of plants and soil properties in shaping the below‐ground microbiome of tropical islands and highlights that understanding the island biogeography of soil microbes requires an integrated analysis of both direct and indirect effects in tropical island ecosystems. Nonetheless, to assess the broader applicability of our findings, future studies should evaluate the generalizability of these patterns across island systems in other climatic regions and well‐designed manipulation experiments should also be considered to reveal the driving mechanism underlying the species‐area relationship precisely.

## Author Contributions

**Yikang Cheng:** conceptualization (lead), formal analysis (lead), investigation (equal), methodology (supporting), validation (lead), visualization (lead), writing – original draft (lead), writing – review and editing (lead). **Yawen Huang:** investigation (equal). **Run Zhang:** investigation (equal). **Shurong Zhou:** data curation (lead), funding acquisition (lead), investigation (lead), supervision (lead), writing – review and editing (equal).

## Funding

This work was supported by the National Natural Science Foundation of China, U22A20449; Hainan Province Science and Technology Special Fund, ZDYF2022SHFZ320, and Collaborative Innovation Center of Ecological Civilization, Hainan University (XTCX2022STC25).

## Conflicts of Interest

The authors declare no conflicts of interest.

## Supporting information


**Data S1:** ece372548‐sup‐0001‐supinfo.docx.

## Data Availability

All data that support the findings of this study have been deposited to the Dryad Digital Repository: https://doi.org/10.5061/dryad.905qfttwt.

## References

[ece372548-bib-0001] Aguirre‐Gutiérrez, J. , S. W. Rifai , X. J. Deng , et al. 2025. “Canopy Functional Trait Variation Across Earth's Tropical Forests.” Nature 641: 129–136. 10.1038/s41586-025-08663-2. 40044867 PMC12043511

[ece372548-bib-0002] Badri, D. V. , and J. M. Vivanco . 2009. “Regulation and Function of Root Exudates.” Plant, Cell and Environment 32: 666–681. 10.1111/j.1365-3040.2008.01926.x 19143988

[ece372548-bib-0003] Bakker, M. G. , L. Otto‐Hanson , A. J. Lange , J. M. Bradeen , and L. L. Kinkel . 2013. “Plant Monocultures Produce More Antagonistic Soil Streptomyces Communities Than High‐Diversity Plant Communities.” Soil Biology and Biochemistry 65: 304–312.

[ece372548-bib-0004] Baldrian, P. , R. López‐Mondéjar , and P. Kohout . 2023. “Forest Microbiome and Global Change.” Nature Reviews Microbiology 21: 487–501. 36941408 10.1038/s41579-023-00876-4

[ece372548-bib-0005] Bardgett, R. D. 2017. “Plant Trait‐Based Approaches for Interrogating Belowground Function.” Biology and Environment 117B: 1–13.

[ece372548-bib-0006] Bardgett, R. D. , and W. H. van der Putten . 2014. “Belowground Biodiversity and Ecosystem Functioning.” Nature 515: 505–511. 25428498 10.1038/nature13855

[ece372548-bib-0007] Bardgett, R. D. , and D. A. Wardle . 2010. Aboveground–Belowground Linkages: Biotic Interactions, Ecosystem Processes, and Global Change. Oxford University Press.

[ece372548-bib-0008] Barton, K. 2014. “MuMIn: Multi‐Model Inference.” R package version, 1.40.0.

[ece372548-bib-0009] Bergmann, J. , A. Weigelt , F. van der Plas , et al. 2020. “The Fungal Collaboration Gradient Dominates the Root Economics Space in Plants.” Science Advances 6: eaba3756. 32937432 10.1126/sciadv.aba3756 PMC7458448

[ece372548-bib-0010] Bolger, A. M. , L. Marc , and U. Bjoern . 2014. “Trimmomatic: A Flexible Trimmer for Illumina Sequence Data.” Bioinformatics 30: 2114–2120. 24695404 10.1093/bioinformatics/btu170 PMC4103590

[ece372548-bib-0011] Cheng, Y. K. , G. Rutten , X. Liu , et al. 2023. “Host Plant Height Explains the Effect of Nitrogen Enrichment on Arbuscular Mycorrhizal Fungal Communities.” New Phytologist 240: 399–411. 37482960 10.1111/nph.19140

[ece372548-bib-0012] Connor, E. F. , and E. D. McCoy . 1979. “The Statistics and Biology of the Species–Area Relationship.” American Naturalist 113: 791–833.

[ece372548-bib-0013] Dassen, S. , R. Cortois , H. Martens , et al. 2017. “Differential Responses of Soil Bacteria, Fungi, Archaea and Protists to Plant Species Richness and Plant Functional Group Identity.” Molecular Ecology 26: 4085–4098. 28489329 10.1111/mec.14175

[ece372548-bib-0014] de Vries, F. T. , P. Manning , J. R. B. Tallowin , et al. 2012. “Abiotic Drivers and Plant Traits Explain Landscape‐Scale Patterns in Soil Microbial Communities.” Ecology Letters 15: 1230–1239. 22882451 10.1111/j.1461-0248.2012.01844.x

[ece372548-bib-0015] Engelhardt, I. C. , A. Welty , S. J. Blazewicz , et al. 2018. “Depth Matters: Effects of Precipitation Regime on Soil Microbial Activity Upon Rewetting of a Plant‐Soil System.” ISME Journal 12: 1061–1071. 29476139 10.1038/s41396-018-0079-z PMC5864200

[ece372548-bib-0016] Ewers, R. M. , and R. K. Didham . 2006. “Confounding Factors in the Detection of Species Responses to Habitat Fragmentation.” Biological Reviews 81: 117–142. 16318651 10.1017/S1464793105006949

[ece372548-bib-0017] Feng, Y. Z. , J. W. Zhang , M. Berdugo , et al. 2022. “Temperature Thresholds Drive the Global Distribution of Soil Fungal Decomposers.” Global Change Biology 28: 2779–2789. 35064621 10.1111/gcb.16096

[ece372548-bib-0018] Ferreira‐Arruda, T. , N. R. Guerrero‐Ramírez , P. Denelle , P. Weigelt , M. Kleyer , and H. Kreft . 2022. “Island Area and Historical Geomorphological Dynamics Shape Multifaceted Diversity of Barrier Island Floras.” Ecography 8: e06238.

[ece372548-bib-0019] Franklin, J. , J. M. Serra‐Diaz , A. D. Syphard , and H. M. Regan . 2016. “Global Change and Terrestrial Plant Community Dynamics.” Proceedings of the National Academy of Sciences of the United States of America 113: 3725–3734. 26929338 10.1073/pnas.1519911113 PMC4833242

[ece372548-bib-0020] Gillespie, L. M. , S. Hattenschwiler , A. Milcu , J. Wambsganss , A. Shihan , and N. Fromin . 2021. “Tree Species Mixing Affects Soil Microbial Functioning Indirectly via Root and Litter Traits and Soil Parameters in European Forests.” Functional Ecology 35: 2190–2204.

[ece372548-bib-0021] Glassman, S. I. , I. J. Wang , and T. D. Bruns . 2017. “Environmental Filtering by pH and Soil Nutrients Drives Community Assembly in Fungi at Fine Spatial Scales.” Molecular Ecology 26: 6960–6973. 29113014 10.1111/mec.14414

[ece372548-bib-0022] Gould, I. J. , J. N. Quinton , A. Weigelt , G. B. De Deyn , and R. D. Bardgett . 2016. “Plant Diversity and Root Traits Benefit Physical Properties Key to Soil Function in Grasslands.” Ecology Letters 19: 1140–1149. 27459206 10.1111/ele.12652 PMC4988498

[ece372548-bib-0023] Grömping, U. 2006. “Relative Importance for Linear Regression in R: The Package Relaimpo.” Journal of Statistical Software 17: 1–27.

[ece372548-bib-0024] Guo, X. , J. J. Feng , Z. Shi , et al. 2018. “Climate Warming Leads to Divergent Succession of Grassland Microbial Communities.” Nature Climate Change 8: 813–818.

[ece372548-bib-0025] Hartemink, A. E. , and N. J. Barrow . 2023. “Soil pH—Nutrient Relationships: The Diagram.” Plant and Soil 486: 209–215.

[ece372548-bib-0026] He, D. , W. J. Shen , J. Eberwein , Q. Zhao , L. J. Ren , and Q. L. L. Wu . 2017. “Diversity and Co‐Occurrence Network of Soil Fungi Are More Responsive Than Those of Bacteria to Shifts in Precipitation Seasonality in a Subtropical Forest.” Soil Biology & Biochemistry 115: 499–510.

[ece372548-bib-0027] Helmus, M. R. , D. L. Mahler , and J. B. Losos . 2014. “Island Biogeography of the Anthropocene.” Nature 513: 543–546. 25254475 10.1038/nature13739

[ece372548-bib-0028] Hennecke, J. , L. Bassi , C. Albracht , et al. 2025. “Plant Species Richness and the Root Economics Space Drive Soil Fungal Communities.” Ecology Letters 28: e70032. 39737799 10.1111/ele.70032 PMC11687415

[ece372548-bib-0029] Kaisermann, A. , P. A. Maron , L. Beaumelle , and J. C. Lata . 2015. “Fungal Communities Are More Sensitive Indicators to Non‐Extreme Soil Moisture Variations Than Bacterial Communities.” Applied Soil Ecology 86: 158–164.

[ece372548-bib-0030] Kong, D. , J. Wang , H. Wu , et al. 2019. “Nonlinearity of Root Trait Relationships and the Root Economics Spectrum.” Nature Communications 10: 2203. 10.1038/s41467-019-10245-6 PMC6525182 31101818

[ece372548-bib-0031] Laliberté, E. , and P. Legendre . 2010. “A Distance‐Based Framework for Measuring Functional Diversity From Multiple Traits.” Ecology 91: 299–305. 20380219 10.1890/08-2244.1

[ece372548-bib-0032] Lefcheck, J. S. 2016. “PIECEWISESEM: Piecewise Structural Equation Modelling in R for Ecology, Evolution, and Systematics.” Methods in Ecology and Evolution 7: 573–579.

[ece372548-bib-0033] Legay, N. , C. Baxendale , K. Gigulis , et al. 2014. “Contribution of Above and Below‐Ground Plant Traits to the Structure and Function of Grassland Soil Microbial Communities.” Annals of Botany 114: 10111021. 10.1093/aob/mcu169 PMC4171078 25122656

[ece372548-bib-0034] Leo, B. 2001. “Random Forests.” Machine Learning 45: 5–32.

[ece372548-bib-0035] Li, S. P. , P. Wang , Y. Chen , et al. 2020. “Island Biogeography of Soil Bacteria and Fungi: Similar Patterns, but Different Mechanisms.” ISME Journal 14: 1886–1896. 32341471 10.1038/s41396-020-0657-8 PMC7305213

[ece372548-bib-0036] Liaw, A. , and M. Wiener . 2002. “Classification and Regression by randomForest.” R News 2: 18–22.

[ece372548-bib-0037] Liu, L. , K. Zhu , N. Wurzburger , and J. Zhang . 2020. “Relationships Between Plant Diversity and Soil Microbial Diversity Vary Across Taxonomic Groups and Spatial Scales.” Ecosphere 11: e02999.

[ece372548-bib-0038] López‐Angulo, J. , M. de la Cruz , J. Chacón‐Labella , et al. 2020. “The Role of Root Community Attributes in Predicting Soil Fungal and Bacterial Community Patterns.” New Phytologist 228: 1070–1082. 32557640 10.1111/nph.16754

[ece372548-bib-0039] MacArthur, R. H. , and E. O. Wilson . 1967. The Theory of Island Biogeography. Princeton University Press.

[ece372548-bib-0040] Mago, T. , and S. L. Salzberg . 2011. “Flash: Fast Length Adjustment of Short Reads to Improve Genome Assemblies.” Bioinformatics 27: 2957–2963. 21903629 10.1093/bioinformatics/btr507 PMC3198573

[ece372548-bib-0041] Martinez‐Ramos, M. , and E. R. Alvarez‐Buylla . 1998. “How Old Are Tropical Rain Forest Trees?” Trends in Plant Science 3: 400–405.

[ece372548-bib-0042] Metze, D. , J. Schnecker , C. L. N. de Carlan , et al. 2024. “Soil Warming Increases the Number of Growing Bacterial Taxa but Not Their Growth Rates.” Science Advances 10: eadk6295. 38394199 10.1126/sciadv.adk6295 PMC10889357

[ece372548-bib-0043] Millard, P. , and B. Singh . 2010. “Does Grassland Vegetation Drive Soil Microbial Diversity?” Nutrient Cycling in Agroecosystems 88: 147–158.

[ece372548-bib-0044] Minasny, B. , S. Y. Hong , A. E. Hartemink , Y. H. Kim , and S. S. Kang . 2016. “Soil pH Increase Under Paddy in South Korea Between 2000 and 2012.” Agriculture, Ecosystems & Environment 221: 205–213.

[ece372548-bib-0045] Ottaviani, G. , G. Keppel , L. Götzenberger , et al. 2020. “Linking Plant Functional Ecology to Island Biogeography.” Trends in Plant Science 25: 329–339. 31953170 10.1016/j.tplants.2019.12.022

[ece372548-bib-0046] Pietikäinen, J. , M. Pettersson , and E. Bååth . 2005. “Comparison of Temperature Effects on Soil Respiration and Bacterial and Fungal Growth Rates.” FEMS Microbiology Ecology 52: 49–58. 16329892 10.1016/j.femsec.2004.10.002

[ece372548-bib-0047] Porazinska, D. L. , E. C. Farrer , M. J. Spasojevic , et al. 2018. “Plant Diversity and Density Predict Belowground Diversity and Function in an Early Successional Alpine Ecosystem.” Ecology 99: 1942–1952. 30024640 10.1002/ecy.2420

[ece372548-bib-0048] Prada‐Salcedo, L. D. , K. Goldmann , A. Heintz‐Buschart , et al. 2021. “Fungal Guilds and Soil Functionality Respond to Tree Community Traits Rather Than to Tree Diversity in European Forests.” Molecular Ecology 30: 572–591. 33226697 10.1111/mec.15749

[ece372548-bib-0049] Reich, P. B. 2014. “The World‐Wide ‘Fast‐Slow’ Plant Economics Spectrum: A Traits Manifesto.” Journal of Ecology 102: 275–301.

[ece372548-bib-0050] Ren, C. , F. Zhao , Z. Shi , et al. 2017. “Differential Responses of Soil Microbial Biomass and Carbon‐Degrading Enzyme Activities to Altered Precipitation.” Soil Biology and Biochemistry 115: 1–10.

[ece372548-bib-0051] Schlatter, D. C. , M. G. Bakker , J. M. Bradeen , and L. L. Kinkel . 2015. “Plant Community Richness and Microbial Interactions Structure Bacterial Communities in Soil.” Ecology 96: 134–142. 26236898 10.1890/13-1648.1

[ece372548-bib-0052] Schloss, P. D. , S. L. Westcott , T. Ryabin , et al. 2009. “Introducing Mothur: Open‐Source, Platform‐Independent, Community‐Supported Software for Describing and Comparing Microbial Communities.” Applied and Environmental Microbiology 75: 7537–7541. 19801464 10.1128/AEM.01541-09 PMC2786419

[ece372548-bib-0053] Schrader, J. , M. Westoby , I. J. Wright , and H. Kreft . 2021. “Disentangling Direct and Indirect Effects of Island Area on Plant Functional Trait Distributions.” Journal of Biogeography 48: 2098–2110.

[ece372548-bib-0054] Shen, C. , A. Gunina , Y. Luo , et al. 2020. “Contrasting Patterns and Drivers of Soil Bacterial and Fungal Diversity Across a Mountain Gradient.” Environmental Microbiology 22: 3287–3301. 32436332 10.1111/1462-2920.15090

[ece372548-bib-0056] Shipley, B. 2009. “Confirmatory Path Analysis in a Generalized Multilevel Context.” Ecology 90: 363–368. 19323220 10.1890/08-1034.1

[ece372548-bib-0055] Shipley, B. 2013. “The AIC Model Selection Method Applied to Path Analytic Models Compared Using a d–Separation Test.” Ecology 94: 560–564. 23687881 10.1890/12-0976.1

[ece372548-bib-0057] Spitzer, C. M. , B. Lindahl , D. A. Wardle , et al. 2021. “Root Trait‐Microbial Relationships Across Tundra Plant Species.” New Phytologist 229: 1508–1520. 33007155 10.1111/nph.16982 PMC7821200

[ece372548-bib-0058] Steinauer, K. , D. Tilman , P. D. Wragg , et al. 2015. “Plant Diversity Effects on Soil Microbial Functions and Enzymes Are Stronger Than Warming in a Grassland Experiment.” Ecology 96: 99–112. 26236895 10.1890/14-0088.1

[ece372548-bib-0059] Strobl, C. , A. L. Boulesteix , A. Zeileis , and T. Hothorn . 2007. “Bias in Random Forest Variable Importance Measures: Illustrations, Sources and a Solution.” BMC Bioinformatics 8: 25. 17254353 10.1186/1471-2105-8-25 PMC1796903

[ece372548-bib-0060] Tedersoo, L. , M. Bahram , S. Põlme , et al. 2014. “Fungal Biogeography. Global Diversity and Geography of Soil Fungi.” Science 346: 1256688. 25430773 10.1126/science.1256688

[ece372548-bib-0061] Wang, C. , and Y. Kuzyakov . 2024. “Mechanisms and Implications of Bacterial‐Fungal Competition for Soil Resources.” ISME Journal 18: wrae073. 38691428 10.1093/ismejo/wrae073 PMC11104273

[ece372548-bib-0062] Wang, C. , L. Ma , X. Zuo , et al. 2022. “Plant Diversity Has Stronger Linkage With Soil Fungal Diversity Than With Bacterial Diversity Across Grasslands of Northern China.” Global Ecology and Biogeography 31: 886–900.

[ece372548-bib-0063] Wang, J. T. , Y. M. Zheng , H. W. Hu , et al. 2016. “Coupling of Soil Prokaryotic Diversity and Plant Diversity Across Latitudinal Forest Ecosystems.” Scientific Reports 6: 19561. 26781165 10.1038/srep19561 PMC4726043

[ece372548-bib-0064] Wang, P. , S. P. Li , X. Yang , J. Zhou , W. Shu , and L. Jiang . 2020. “Mechanisms of Soil Bacterial and Fungal Community Assembly Differ Among and Within Islands.” Environmental Microbiology 22: 1559–1571. 31736222 10.1111/1462-2920.14864

[ece372548-bib-0065] Wardle, D. A. , R. D. Bardgett , J. N. Klironomos , H. Setälä , W. H. van der Putten , and D. H. Wall . 2004. “Ecological Linkages Between Aboveground and Belowground Biota.” Science 304: 1629–1633. 15192218 10.1126/science.1094875

[ece372548-bib-0066] Whittaker, R. J. , J. M. Fernández‐Palacios , T. J. Matthews , M. K. Borregaard , and K. A. Triantis . 2017. “Island Biogeography: Taking the Long View of Nature's Laboratories.” Science 57: eaam8326. 10.1126/science.aam8326 28860356

[ece372548-bib-0067] Wright, I. J. , P. B. Reich , M. Westoby , et al. 2004. “The Worldwide Leaf Economics Spectrum.” Nature 428: 821–827. 15103368 10.1038/nature02403

[ece372548-bib-0068] Wu, L. , Y. Zhang , X. Guo , et al. 2022. “Reduction of Microbial Diversity in Grassland Soil Is Driven by Long‐Term Climate Warming.” Nature Microbiology 7: 1054–1062. 10.1038/s41564-022-01147-3 35697795

[ece372548-bib-0069] Xu, H. , L. Huang , J. Chen , et al. 2023. “Changes in Soil Microbial Activity and Their Linkages With Soil Carbon Under Global Warming.” Catena 232: 107419.

[ece372548-bib-0070] Xu, M. S. , A. N. Yang , X. D. Yang , et al. 2023. “Island Area and Remoteness Shape Plant and Soil Bacterial Diversity Through Land Use and Biological Invasion.” Functional Ecology 37: 1232–1244.

[ece372548-bib-0071] Yang, X. , K. Zhu , M. E. Loik , and W. Sun . 2021. “Differential Responses of Soil Bacteria and Fungi to Altered Precipitation in a Meadow Steppe.” Geoderma 384: 114812.

[ece372548-bib-0072] Yu, Q. S. , C. Q. He , M. A. Anthony , et al. 2024. “Decoupled Responses of Plants and Soil Biota to Global Change Across the World's Land Ecosystems.” Nature Communications 15: 10369. 10.1038/s41467-024-54304-z PMC11605088 39609374

[ece372548-bib-0073] Zheng, Y. , P. Maitra , H. Y. Gan , et al. 2021. “Soil Fungal Diversity and Community Assembly: Affected by Island Size or Type?” FEMS Microbiology Ecology 97: fiab062. 33890666 10.1093/femsec/fiab062

[ece372548-bib-0074] Zhou, S. R. , H. Qin , R. F. Liao , and Y. K. Cheng . 2024. “Habitat Quality Drives the Species‐Area Relationship of Plants and Soil Microbes in an Ocean Archipelago.” Oikos 11: e10660.

[ece372548-bib-0075] Zhou, Z. , C. Wang , and Y. Luo . 2020. “Meta‐Analysis of the Impacts of Global Change Factors on Soil Microbial Diversity and Functionality.” Nature Communications 11: 3072. 10.1038/s41467-020-16881-7 PMC7300008 32555185

