# Supplementary material for: Disentangling Drivers of Soil Bacterial and Fungal Diversity on Tropical Islands
Source: Ecol Evol. 2025 Nov 23;15(11):e72548. doi: 10.1002/ece3.72548 (PMC12640883; doi:10.1002/ece3.72548)

*Supplementary Information for*

[**Disentangling drivers of soil bacteria and fungi**](https://www.sciencedirect.com/science/article/pii/S0048969721056321) **diversity in** tropical islands

Yikang Cheng^1^, Yawen Huang^2^, Run Zhang^2^, Shurong Zhou^1^*

*^1^ School of Ecology, Hainan University, Haikou 570228, P. R. China*

*^2^ School of Tropical Agriculture and Forestry, Hainan University, Haikou 570228, P. R. China*

**Author e-mails:**

Yikang Cheng: [ykcheng@hainanu.edu.cn](mailto:ykcheng@hainanu.edu.cn)

Yawen Huang: [yara678678@163.com](mailto:yara678678@163.com)

Run Zhang: zhrun0408@163.com

Shurong Zhou: [zhshrong@hainanu.edu.cn](mailto:zhshrong@hainanu.edu.cn)

**ORCID information:**

Shurong Zhou: <http://orcid.org/0000-0002-7093-1703>

Yikang Cheng: [https://orcid.org/0000-0003-4942-3904](https://orcid.org/0000-0003-4942-3904%20)

***Corresponding author:** Shurong Zhou, E-mail: [zhshrong@hainanu.edu.cn](mailto:zhshrong@hainanu.edu.cn)

**Table S1** Basic Information on 20 study islands.

| **Island** | **Area (ha)** | **Number of plots** | **MAT (℃)** | **MAP (mm)** | **STN** | **STP** | **SOC** | **SCN** | **STK** | **Soil salinity** | **Soil pH** |
| --- | --- | --- | --- | --- | --- | --- | --- | --- | --- | --- | --- |
| BSZ | 1.19 | 2 | 26.85 | 1535 | 0.03 | 0.17 | 0.17 | 6.50 | 0.12 | 0.74 | 8.38 |
| WZD | 1.51 | 2 | 25.09 | 1616 | 0.30 | 0.67 | 3.96 | 13.37 | 5.37 | 1.09 | 5.15 |
| ZSZ | 3.24 | 2 | 26.85 | 1535 | 0.03 | 0.21 | 0.18 | 6.15 | 0.11 | 0.49 | 8.24 |
| NSZ | 5.27 | 3 | 26.85 | 1535 | 0.05 | 0.40 | 0.44 | 8.41 | 0.11 | 0.41 | 8.04 |
| XGD | 6.92 | 3 | 25.30 | 1340 | 0.77 | 1.84 | 7.92 | 10.58 | 3.10 | 0.81 | 7.31 |
| GZD | 8.12 | 3 | 25.08 | 1601 | 0.18 | 0.26 | 2.14 | 11.70 | 4.00 | 0.29 | 6.02 |
| ND | 9.76 | 4 | 26.85 | 1535 | 0.07 | 0.53 | 0.70 | 9.09 | 0.12 | 0.89 | 8.34 |
| ZD | 10.25 | 3 | 26.84 | 1496 | 0.16 | 9.12 | 1.66 | 10.42 | 0.01 | 0.83 | 7.52 |
| DLD | 13.45 | 4 | 25.32 | 1375 | 0.17 | 0.32 | 1.83 | 10.55 | 5.35 | 0.34 | 7.33 |
| JJD | 16.63 | 4 | 25.08 | 1561 | 0.24 | 0.47 | 3.07 | 12.88 | 4.80 | 0.44 | 6.42 |
| JQD | 20.94 | 5 | 27.05 | 1472 | 0.28 | 26.58 | 2.93 | 10.42 | 0.12 | 0.79 | 7.46 |
| XSZ | 21.65 | 4 | 26.84 | 1524 | 0.04 | 0.18 | 0.28 | 7.97 | 0.02 | 0.51 | 8.10 |
| BAD | 25.47 | 4 | 25.08 | 1601 | 0.32 | 0.59 | 3.67 | 11.51 | 0.75 | 0.46 | 6.54 |
| ZS | 25.58 | 5 | 26.82 | 1541 | 0.37 | 20.70 | 4.22 | 10.98 | 0.12 | 0.92 | 7.18 |
| BD | 28.20 | 6 | 26.31 | 1501 | 0.09 | 1.47 | 0.93 | 10.80 | 0.01 | 0.41 | 7.80 |
| GQD | 32.15 | 6 | 27.05 | 1472 | 0.31 | 36.04 | 3.27 | 10.79 | 0.12 | 0.73 | 7.49 |
| FJZD | 35.84 | 4 | 25.11 | 1537 | 0.19 | 0.24 | 2.40 | 12.59 | 6.25 | 0.24 | 5.67 |
| ZZD | 43.61 | 5 | 25.73 | 1610 | 0.19 | 0.24 | 2.22 | 11.62 | 5.46 | 0.25 | 5.75 |
| WZZ | 102.51 | 6 | 26.13 | 1429 | 0.24 | 0.54 | 2.55 | 10.32 | 4.35 | 0.29 | 6.29 |
| DZD | 405.99 | 8 | 24.57 | 1554 | 0.21 | 0.19 | 2.62 | 12.80 | 2.23 | 0.42 | 6.35 |

* The sampled islands are ranked according to their area (from smallest to largest). MAP: mean annual precipitation; MAT: mean annual temperature; SOC: soil organic carbon content; STN: soil total nitrogen content; STP: soil total phosphorus content; STK: soil total potassium content; SCN: soil carbon/nitrogen ratio.

**Table S2** The influences of climate and island area on abiotic (i.e., soil properties) and biotic variables (i.e., plant richness and functional diversity). Significant relationships are highlighted in bold (*P* < 0.05). CWM: community weighted mean; FD_is_: functional dispersion; SLA: specific leaf area; LDMC: leaf dry matter content; SOC: soil organic carbon content; STN: soil total nitrogen content; STP: soil total phosphorus content; STK: soil total potassium content; SCN: soil carbon/nitrogen ratio. The blue background color indicates a significant impact (*P < 0.05*).

|  | **Mean annual temperature** | | | **Mean annual** **precipitation** | | | **Island area** | | |
| --- | --- | --- | --- | --- | --- | --- | --- | --- | --- |
| **Dependent variable** | *Slope* | [*R-square*](https://blog.csdn.net/Sirow/article/details/109630056) | *P-value* | *Slope* | [*R-square*](https://blog.csdn.net/Sirow/article/details/109630056) | *P-value* | *Slope* | [*R-square*](https://blog.csdn.net/Sirow/article/details/109630056) | *P-value* |
| Plant richness | -10.5100 | **0.424** | **< 0.001** | 0.02121 | < 0.001 | 0.348 | 13.8820 | **0.306** | **< 0.001** |
| CWM SLA | 18.9170 | **0.099** | **0.002** | -0.06443 | < 0.001 | 0.424 | 0.8705 | < 0.001 | 0.930 |
| CWM LDMC | -0.1191 | **0.430** | **< 0.001** | -0.00024 | < 0.001 | 0.348 | 0.1186 | **0.171** | **< 0.001** |
| FD_is_ SLA | -0.1235 | **0.195** | **< 0.001** | 0.00001 | < 0.001 | 0.978 | 0.1329 | **0.088** | **0.004** |
| FD_is_ LDMC | -0.1697 | **0.312** | **< 0.001** | 0.00025 | < 0.001 | 0.562 | 0.1434 | **0.084** | **0.005** |
| SOC | -0.5820 | **0.048** | **0.027** | -0.00602 | 0.028 | 0.072 | 0.3042 | < 0.001 | 0.462 |
| STN | -0.0382 | 0.018 | 0.118 | -0.00076 | **0.063** | **0.013** | 0.0176 | < 0.001 | 0.645 |
| STP | 7.4460 | **0.179** | **< 0.001** | -0.03544 | 0.015 | 0.141 | 0.5411 | < 0.001 | 0.856 |
| SCN | -1.3563 | **0.350** | **< 0.001** | 0.00613 | 0.034 | 0.053 | 1.4574 | **0.162** | **< 0.001** |
| STK | -1.8260 | **0.430** | **< 0.001** | 0.00035 | < 0.001 | 0.929 | 0.6201 | 0.001 | 0.194 |
| Soil salinity | 0.1377 | **0.097** | **0.003** | -0.00034 | < 0.001 | 0.562 | -0.1841 | **0.070** | **0.010** |
| Soil pH | 0.7367 | **0.362** | **< 0.001** | -0.00454 | **0.077** | **0.007** | -0.6105 | **0.095** | **0.003** |

**Figure S1** Hypothesized mechanisms for the potential pathways of biotic and abiotic variables on soil microbial diversity (i.e., bacterial and fungal richness). The full structural equation model (SEM) shows a possible causal linkage between climate (including mean annual precipitation and mean annual temperature), island area, above-ground plant richness, community weighted mean (CWM) and functional dispersion (FD_is_) of two plant traits (specific leaf area (SLA) and leaf dry matter content (LDMC)), below-ground soil properties, and soil microbial diversity.


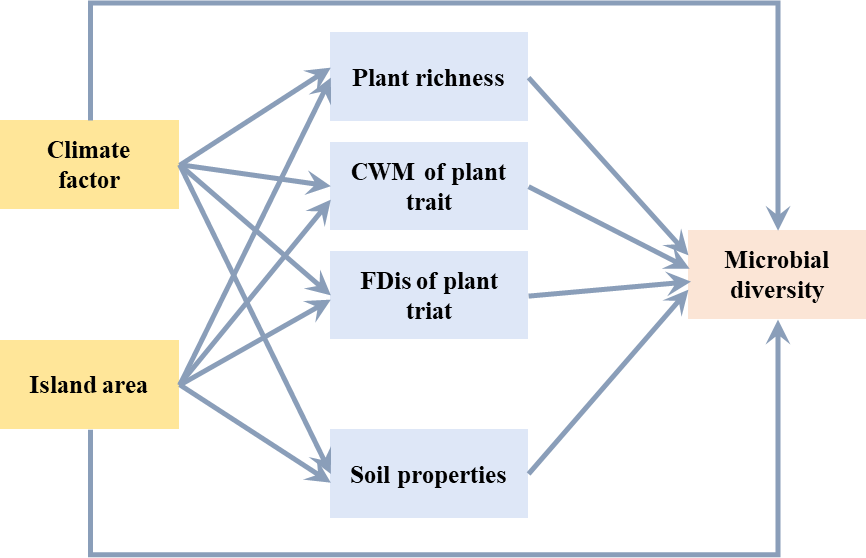


**Figure S2** Location of the study site.


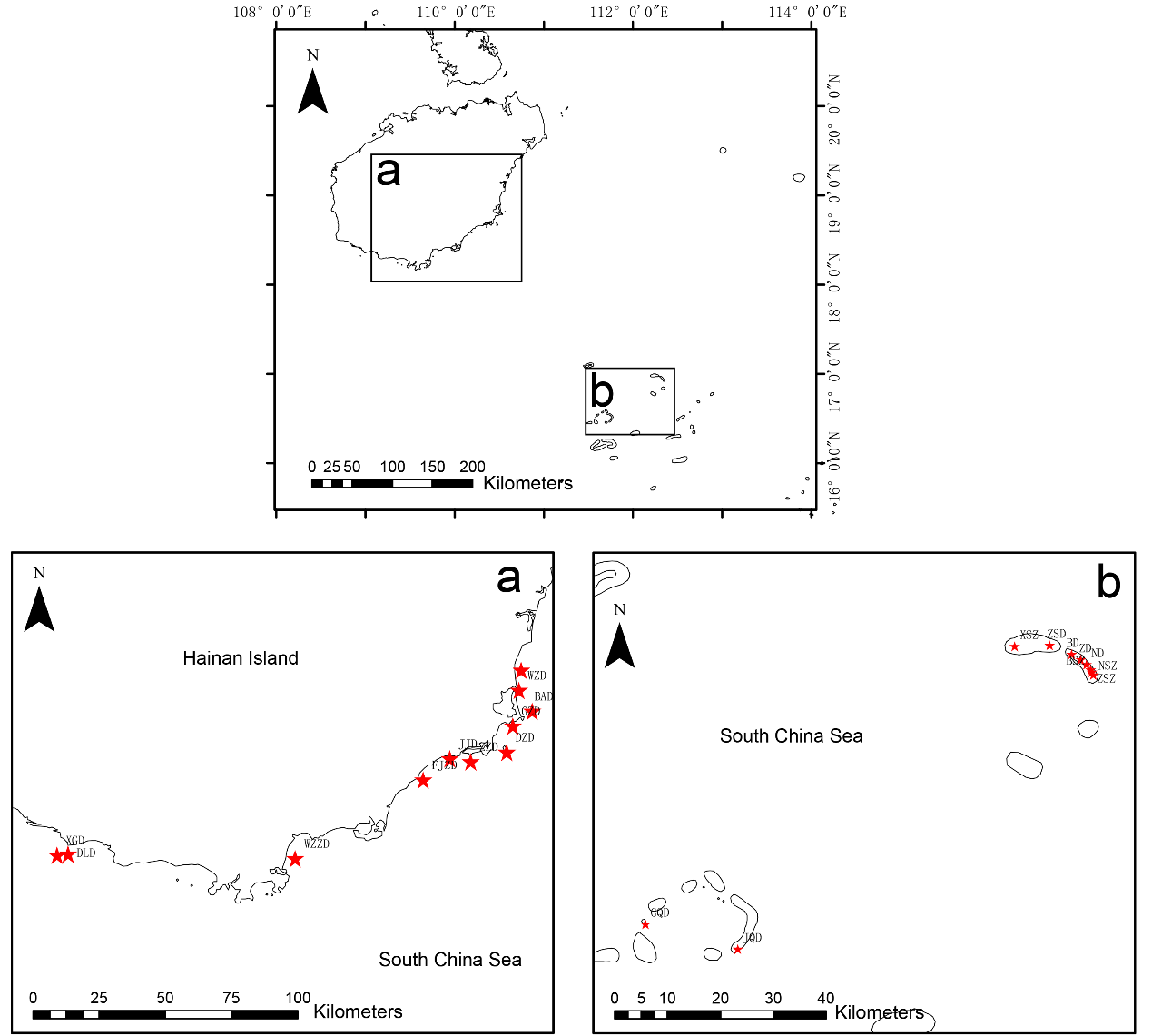


**Figure S3** The correlation between island precipitation and isolation.





**Figure S4** Results of the random forest analysis of the relative importance of abiotic and biotic variables on soil bacterial (a) and fungal (b) richness. MAP: mean annual precipitation; MAT: mean annual temperature; CWM: community weighted mean; FD_is_: functional dispersion; SLA: specific leaf area; LDMC: leaf dry matter content; SOC: soil organic carbon content; STN: soil total nitrogen content; STP: soil total phosphorus content; STK: soil total potassium content; SCN: soil carbon/nitrogen ratio.


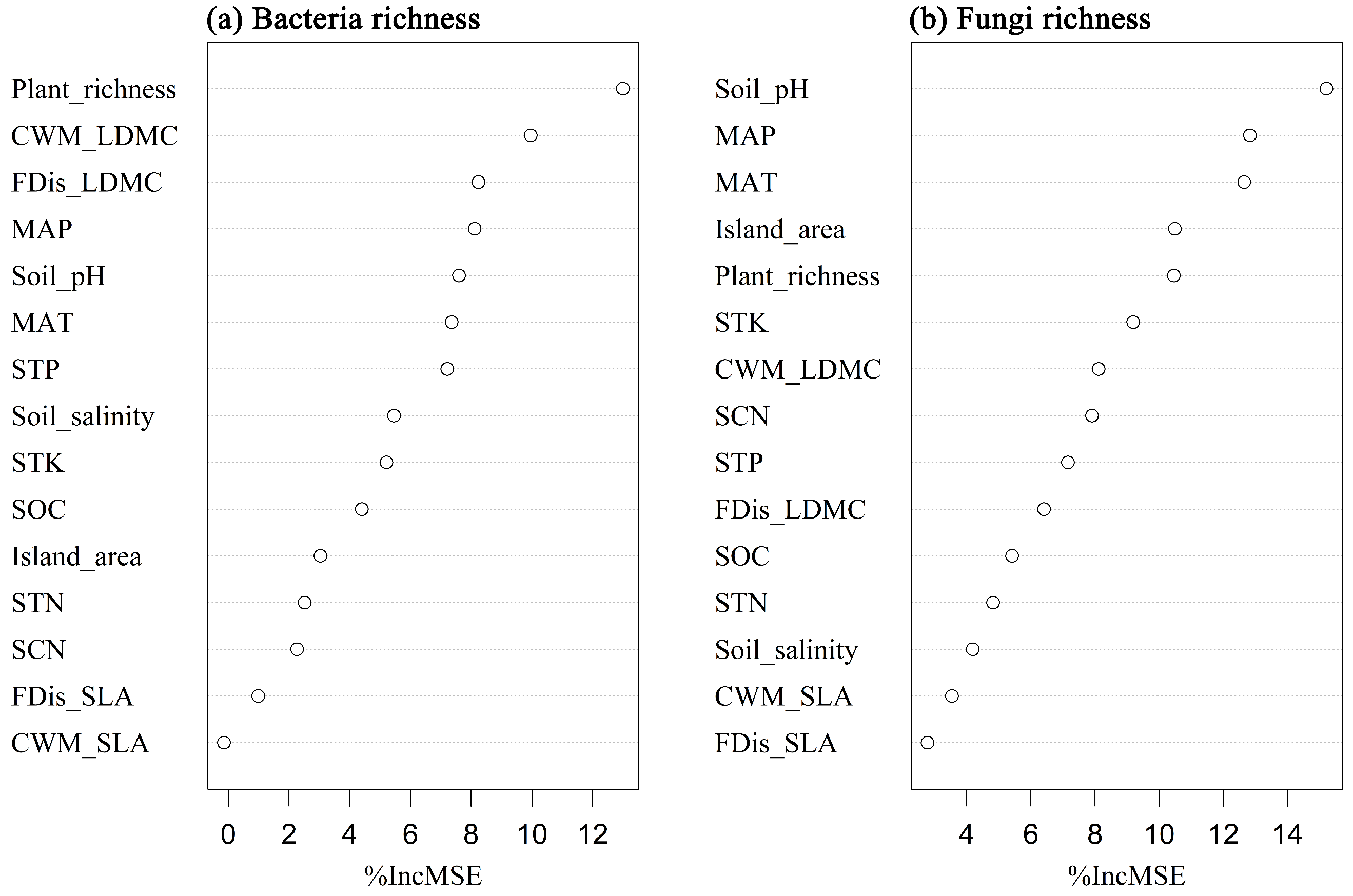


**Figure S5** Pearson’s correlations between the eight most important predictors of the soil bacteria (a) and fungi (b) richness. MAP: mean annual precipitation; MAT: mean annual temperature; CWM: community-weighted mean value; FD_is_: functional dispersion; SLA: specific leaf area; LDMC: leaf dry matter content; SOC: soil organic carbon content; STN: soil total nitrogen content; STP: soil total phosphorus content; STK: soil total potassium content; SCN: soil carbon/nitrogen ratio.


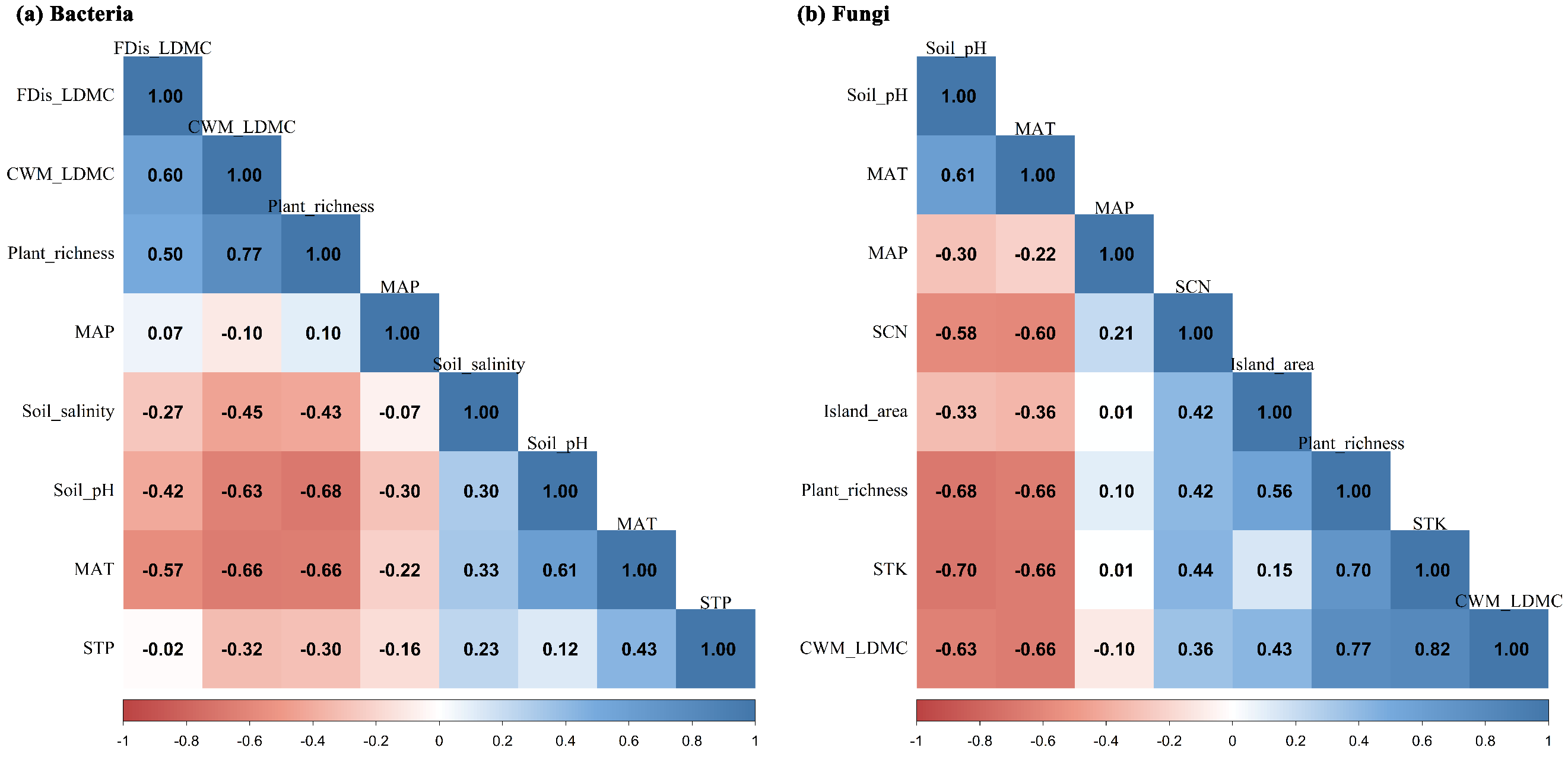


**Figure S6** Results of the initial structural equation model (SEM) showing the pathways of climate variables and island area affecting soil bacterial (a) and fungal (b) richness through aboveground plant community and/or belowground soil properties. The coefficients are standardized prediction coefficients for each causal path. Solid lines represent significant relationships (*P < 0.05*), while dashed lines represent non-significant relationships. Line thickness is proportional to the strength of the relationship. Numbers above and below arrows are standardized path coefficients (significance: *^***^P < 0.001*; *^**^P < 0.01*; *^*^P < 0.05*). *R^2^* represents the proportion of variance explained for each dependent variable. MAP: mean annual precipitation; MAT: mean annual temperature; CWM: community weighted mean; FDis: functional dispersion; SLA: specific leaf area; LDMC: leaf dry matter content; SCN: soil carbon/nitrogen ratio.


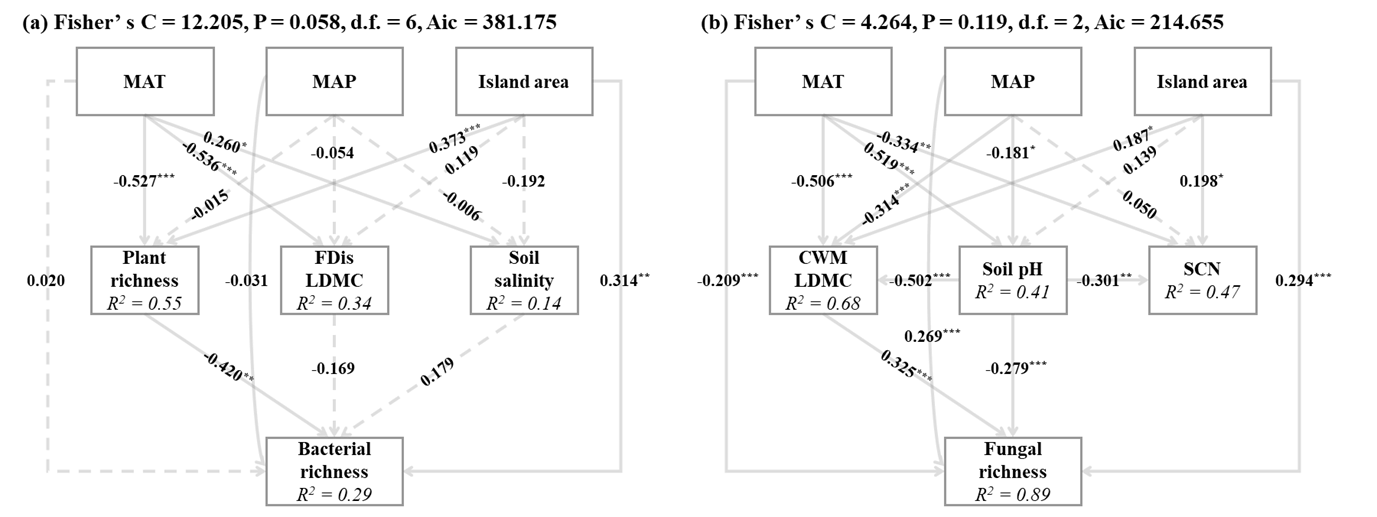

Supplement: Supplementary file 1 — Data S1: ece372548‐sup‐0001‐supinfo.docx. [file ECE3-15-e72548-s001.docx]
